# Artificial intelligence-enhanced electrocardiography for the prediction of future type 2 diabetes mellitus: a model-development and multicentre validation study

**DOI:** 10.1093/ehjdh/ztag118

**Published:** 2026-07-22

**Authors:** Libor Pastika, Konstantinos Patlatzoglou, Ewa Sieliwonczyk, Joseph Barker, Boroumand Zeidaabadi, Kathryn A McGurk, Sandhi M Barreto, Lidyane Camelo, Sadia Khan, William R Scott, Declan P O’Regan, Bruce B Duncan, Maria I Schmidt, James S Ware, Shivani Misra, Daniel B Kramer, Jonathan W Waks, Nicholas S Peters, Antonio Luiz Pinho Ribeiro, Arunashis Sau, Fu Siong Ng

**Affiliations:** National Heart and Lung Institute, Imperial College London, Hammersmith Campus, Du Cane Road, London W12 0NN, UK; Department of Cardiology, Imperial College Healthcare NHS Trust, Hammersmith Hospital, Du Cane Road, London W12 0NN, UK; Chelsea and Westminster Hospital NHS Foundation Trust, 369 Fulham Road, London SW10 9NH, UK; National Heart and Lung Institute, Imperial College London, Hammersmith Campus, Du Cane Road, London W12 0NN, UK; National Heart and Lung Institute, Imperial College London, Hammersmith Campus, Du Cane Road, London W12 0NN, UK; MRC Laboratory of Medical Sciences, Imperial College London, London, UK; National Heart and Lung Institute, Imperial College London, Hammersmith Campus, Du Cane Road, London W12 0NN, UK; National Heart and Lung Institute, Imperial College London, Hammersmith Campus, Du Cane Road, London W12 0NN, UK; National Heart and Lung Institute, Imperial College London, Hammersmith Campus, Du Cane Road, London W12 0NN, UK; MRC Laboratory of Medical Sciences, Imperial College London, London, UK; Department of Preventive Medicine, School of Medicine & Hospital das Clínicas/Empresa Brasileira de Serviços Hospitalares, Universidade Federal de Minas Gerais, Belo Horizonte, Brazil; Department of Preventive Medicine, School of Medicine & Hospital das Clínicas/Empresa Brasileira de Serviços Hospitalares, Universidade Federal de Minas Gerais, Belo Horizonte, Brazil; National Heart and Lung Institute, Imperial College London, Hammersmith Campus, Du Cane Road, London W12 0NN, UK; Chelsea and Westminster Hospital NHS Foundation Trust, 369 Fulham Road, London SW10 9NH, UK; MRC Laboratory of Medical Sciences, Imperial College London, London, UK; Institute of Clinical Sciences, Faculty of Medicine, Imperial College London, London, UK; MRC Laboratory of Medical Sciences, Imperial College London, London, UK; Postgraduate Program in Epidemiology and Hospital de Clínicas de Porto Alegre, Federal University of Rio Grande do Sul, Porto Alegre, Rio Grande do Sul, Brazil; Postgraduate Program in Epidemiology and Hospital de Clínicas de Porto Alegre, Federal University of Rio Grande do Sul, Porto Alegre, Rio Grande do Sul, Brazil; National Heart and Lung Institute, Imperial College London, Hammersmith Campus, Du Cane Road, London W12 0NN, UK; MRC Laboratory of Medical Sciences, Imperial College London, London, UK; Division of Metabolism, Digestion & Reproduction, Imperial College London, London, UK; Department of Diabetes & Endocrinology, Imperial College Healthcare NHS Trust, London, UK; National Heart and Lung Institute, Imperial College London, Hammersmith Campus, Du Cane Road, London W12 0NN, UK; Richard A. and Susan F. Smith Center for Outcomes Research in Cardiology, Beth Israel Deaconess Medical Center, Harvard Medical School, Boston MA USA; Harvard-Thorndike Electrophysiology Institute, Beth Israel Deaconess Medical Center, Harvard Medical School, Boston, MA, USA; National Heart and Lung Institute, Imperial College London, Hammersmith Campus, Du Cane Road, London W12 0NN, UK; Department of Cardiology, Imperial College Healthcare NHS Trust, Hammersmith Hospital, Du Cane Road, London W12 0NN, UK; Department of Internal Medicine, Faculdade de Medicina, and Telehealth Center and Cardiology Service, Hospital das Clínicas (A.L.P.R.), Universidade Federal de Minas Gerais, Belo Horizonte, Brazil; National Heart and Lung Institute, Imperial College London, Hammersmith Campus, Du Cane Road, London W12 0NN, UK; Department of Cardiology, Imperial College Healthcare NHS Trust, Hammersmith Hospital, Du Cane Road, London W12 0NN, UK; National Heart and Lung Institute, Imperial College London, Hammersmith Campus, Du Cane Road, London W12 0NN, UK; Department of Cardiology, Imperial College Healthcare NHS Trust, Hammersmith Hospital, Du Cane Road, London W12 0NN, UK; Chelsea and Westminster Hospital NHS Foundation Trust, 369 Fulham Road, London SW10 9NH, UK

**Keywords:** Artificial intelligence (AI), Electrocardiography (ECG), Type 2 diabetes mellitus (T2DM), Diabetes prediction, AI-enhanced ECG, AI-ECG, Risk stratification, Pre-diabetes, AIRE platform

## Abstract

**Aims:**

A significant proportion of type 2 diabetes cases remain undiagnosed despite screening advances, carrying substantial cardiometabolic risk. Artificial intelligence-enhanced electrocardiography (AI-ECG) detects subtle ECG changes in subclinical disease, potentially enabling opportunistic screening.

**Methods and results:**

We developed AI-ECG Risk Estimator for Diabetes Mellitus (AIRE-DM), a convolutional neural network with discrete-time survival loss, for diagnosis of prevalent and prediction of incident type 2 diabetes. It was trained on 1 163 401 ECGs from 189 537 individuals from Beth Israel Deaconess Medical Center (BIDMC) and externally validated in UK Biobank (UKB; *n* = 65 606) and ELSA-Brasil (*n* = 13 739). AI-ECG Risk Estimator for Diabetes Mellitus demonstrated moderate discrimination for prevalent type 2 diabetes (area under the receiver operating characteristic curve: BIDMC 0.724, UKB 0.733, ELSA-Brasil 0.706) and incident type 2 diabetes (C-index: BIDMC 0.667, UKB 0.688, ELSA-Brasil 0.625). The highest AIRE-DM risk quartile had elevated incident diabetes risk vs. the lowest (hazard ratio: BIDMC 4.75, UKB 7.52, ELSA-Brasil 3.96). AI-ECG Risk Estimator for Diabetes Mellitus was non-inferior to the American Diabetes Association Diabetes Risk Test in BIDMC, with improved predictive accuracy when combined. In normoglycaemic patients, AIRE-DM was superior to glycated haemoglobin (HbA1c) for predicting incident diabetes in BIDMC and non-inferior in ELSA-Brasil. The highest risk quartile reached 5% cumulative type 2 diabetes mellitus incidence 5.4 years (BIDMC) and 4.8 years (ELSA-Brasil) earlier than the lowest risk quartile, after adjusting for HbA1c, age, and sex. Phenome- and genome-wide association studies revealed biologically plausible associations with glucose regulation, cardiac morphology, diastolic dysfunction, arterial stiffness, and lipid metabolism.

**Conclusion:**

AI-ECG Risk Estimator for Diabetes Mellitus detects prevalent type 2 diabetes and predicts incident disease, uniquely identifying high-risk individuals within the normoglycaemic range. Combined with clinical scores or biomarkers, it enhances risk stratification, enabling earlier intervention.

## Graphical Abstract

**Graphical Abstract:**
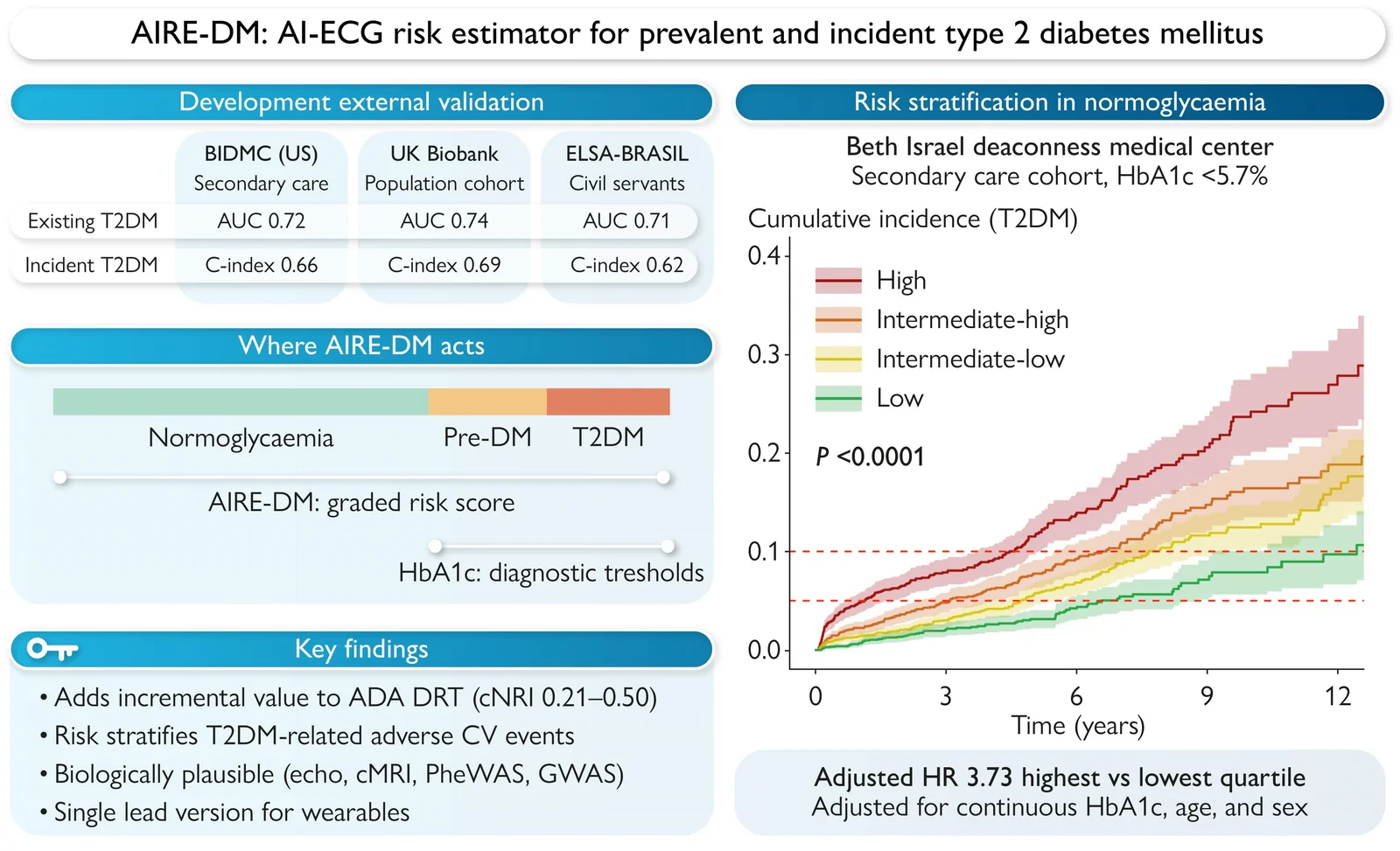

## Introduction

Type 2 diabetes mellitus (T2DM) incidence is rising globally, yet a substantial proportion of cases remain undiagnosed.^1^ At the time of clinical diagnosis, a significant proportion of patients already exhibit microvascular complications.^2–4^ Moreover, the incidence rates of both microvascular and macrovascular complications are high among individuals with newly diagnosed T2DM, with a median time to incidence of diabetes-related complication between 3 and 5 years of diagnosis.^2^ These findings suggest that either clinical diagnosis of T2DM is delayed or that a significant proportion of individuals with T2DM experience microvascular and macrovascular complications at an earlier stage of dysglycaemia.

Current screening strategies for T2DM predominantly rely on clinical risk scores based on lifestyle and clinical factors, followed by confirmatory tests such as fasting glucose and glycated haemoglobin (HbA1c) measurements. This glucose-centric approach categorizes individuals with normoglycaemia, pre-diabetes, or diabetes. However, this approach overlooks individuals who are at high risk for progressing to T2DM despite being labelled as normoglycaemic. This reliance on the same biomarker to both define and detect disease works well at population levels but risks missing a critical window for early intervention in higher risk individuals, who may have glucose levels in the normal range but are already insulin resistant and therefore at higher risk of developing T2DM.^5^

The electrocardiogram (ECG) is a routinely performed diagnostic tool that is independent of glycaemic measures—with over 600 million ECGs conducted in Europe^6^ and the USA^7^ alone. Specific ECG signatures have been associated with both T2DM and pre-diabetes,^8–10^ and artificial intelligence-enhanced ECG (AI-ECG) models have demonstrated promising capabilities in predicting future cardiac and non-cardiac diseases in a cost-effective manner.^11–15^ These findings warrant further evaluation to determine the utility of AI-ECG in routine T2DM screening and future risk prediction, given the current limitations of glucose-centric approaches.

In this study, we present the AI-ECG Risk Estimator for Diabetes Mellitus (AIRE-DM), which was evaluated for its effectiveness in identifying both prevalent and incident T2DM. Furthermore, the performance of AIRE-DM was compared with that of an existing T2DM screening modality, and a series of analyses were conducted to establish the biological plausibility of its predictions. This study aims to assess whether a non-glucose centric measurement, such as the ECG, could complement existing screening strategies and potentially address current gaps for the opportunistic early detection of T2DM.

## Methods

### Ethical approvals

This study complies with all relevant ethical regulations; further details are provided in the Supplementary Methods (p3).

### Cohorts

We examined three cohorts: the Beth Israel Deaconess Medical Center (BIDMC) cohort, a secondary care dataset from Boston, USA; the UK Biobank (UKB), a longitudinal population study consisting of healthy volunteers; and the ELSA-Brasil cohort, a longitudinal study of Brazilian public servants. The BIDMC cohort consisted of 1 163 401 ECGs from 189 537 individuals aged 16 and above who underwent ECGs between 2000 and 2023. The UKB cohort comprised 65 610 participants aged 40–69 at enrolment between 2006 and 2010^16^ with available ECGs. Outcome data were linked to cancer and death registries, hospital admissions, and primary care records. ELSA-Brasil comprises 15 105 Brazilian public servants aged 35–74 at enrolment, and 13 739 subjects with ECG and outcome data available for analysis. The full inclusion criteria and protocol have been previously described.^17^ Refer to the Supplementary Methods (pp3–4) for additional details and *Table 1* for cohort demographics.

**Table 1:** Cohort demographics

	BIDMC	UK Biobank	ELSA-Brasil
*n* subjects	189 537	65 610	13 739
Age	55.6 (18.2)	65.4 (7.9)	52.3 (9.1)
Follow-up (years)	5.47 (5.81)	3.01 (2.66)	10.4 (2.9)
Sex (M)	90 791 (47.9)	31 825 (48.5)	6256 (45.5)
Smoker	11 396 (6.0)	1643 (2.5)	1801 (13.1)
Previous HF	4762 (2.5)	518 (0.8)	251 (1.8)
Previous MI	2129 (1.1)	1610 (2.5)	227 (1.7)
Prevalent HTN	53 475 (28.2)	20 673 (31.5)	4949 (36.0)
Prevalent T2DM	22 941 (12.1)	3838 (5.8)	2728 (19.9)
Incident T2DM	27 537 (14.5)	418 (0.6)	1587 (14.5)
Mortality	34 938 (18.4)	790 (1.2)	599 (4.4)

Summary of the key demographic and clinical characteristics across the BIDMC, UK Biobank, and ELSA-Brasil cohorts. Data from the first ECG per subject is provided in the BIDMC dataset. Categorical variables are expressed as *n* (%) and continuous variables as mean (SD).

BIDMC, Beth Israel Deaconess Medical Center; HF, heart failure; HTN, hypertension; MI, myocardial infarction; T2DM, type 2 diabetes mellitus; UKB, UK Biobank.

### Outcome definition

In the BIDMC dataset, T2DM was determined using ICD-9/10 codes. In the UKB dataset, T2DM was defined algorithmically using a combination of ICD codes, self-reported diagnoses, primary care records, and causes of death. In ELSA-Brasil, T2DM was ascertained based on laboratory measurements taken during visits and reports from annual telephone follow-up surveillance. Cases were defined as individuals who either reported a medical diagnosis of T2DM or current use of diabetes medications, or who had laboratory values exceeding thresholds for fasting plasma glucose (≥7.0 mmol/L; 126 mg/dL), 2-h post-load glucose (≥11.1 mmol/L; 200 mg/dL), or HbA1c (≥48 mmol/mol; 6.5%).^18^ To minimize the risk of reverse causality, incident T2DM was restricted to those individuals with a minimum follow-up period of 30 days before censoring. Individuals without diabetes were defined as those without any recorded instance of T2DM based on the respective criteria used in each dataset. See the Supplementary Methods for more details.

### Model development

The AIRE-DM model was developed using the BIDMC dataset. To avoid data leakage, the dataset was partitioned based on patient ID, with a 50/10/40% split for derivation, validation, and holdout test sets, respectively. Leads III, aVL, aVR, and aVF were excluded as they are linearly dependent on leads I and II, resulting in an input size of 4096 × 8 for each ECG. In parallel, a single-lead (lead I) version of the AIRE-DM model was trained to ascertain its effectiveness in potential wearable technology.

The model architecture was based on a previously described convolutional neural network that incorporates residual blocks, with a final sigmoid layer.^19^ It was first trained as a classification task for prevalent T2DM, then adapted for future T2DM prediction using a previously described discrete-time survival loss function to accommodate time-to-event and censorship.^20^ The trained weights from the classification model served as the starting parameters for the future T2DM prediction model, rather than random initialization. While all ECGs were used in training, only outpatient ECGs were used for the BIDMC test set as future T2DM prediction is most relevant for outpatients. See the Supplementary Methods and the Code availability sections for more details.

### Clinical risk scores

To evaluate performance of AIRE-DM against existing approaches for screening, we calculated the American Diabetes Association Diabetes Risk Test (ADA DRT), originally developed by Bang *et al*. for the identification of undiagnosed prevalent T2DM,^21^ in participants from the UKB; we employed the original weighted scoring system^21^ in UKB and ELSA-Brasil, which included variables such as age, sex, family history of T2DM, hypertension, body mass index (BMI), and physical inactivity. In the BIDMC cohort, data on family history of T2DM and physical inactivity were not available, so we modified the ADA DRT by subtracting the points associated with these missing variables from the overall score.

### Statistical analyses

We evaluated AIRE-DM’s detection of prevalent T2DM using AUROC and its ability to predict future incident T2DM using the concordance index. The additive value of AIRE-DM was then assessed alongside (i) the modified ADA DRT in the BIDMC cohort, (ii) the ADA DRT in the UKB, and (iii) in ELSA-Brasil. We employed a bootstrap resampling approach to compare the discriminative abilities of AIRE-DM, ADA DRT, and their combination. For each of 1000 bootstrap samples, we trained three models: (i) AIRE-DM alone, (ii) ADA DRT alone, and (iii) the combined model incorporating both AIRE-DM and ADA DRT. The ADA DRT was used as a continuous score (i.e. the sum of the individual parameters). We calculated the performance metrics (AUROC or C-index) for each model on each bootstrap sample. For the survival models, estimates of model performance were optimism-corrected using Harrell’s bias method. The likelihood ratio tests were used to determine if integrating AIRE-DM with ADA DRT significantly improved model fit. Continuous net reclassification index (NRI) analyses were performed to evaluate the classification enhancement provided by AIRE-DM. Additionally, non-nested models were compared using the partial likelihood ratio test for survival models and the Vuong non-nested test for binary models.

Furthermore, AIRE-DM and HbA1c were compared across predefined HbA1c strata in participants with available baseline HbA1c—specifically, BIDMC patients in whom HbA1c was measured within 90 days of the index ECG (*n* = 9244) and ELSA-Brasil participants in whom HbA1c and ECG were recorded on the same day (*n* = 13 717). Glycated haemoglobin was classified according to WHO 2019 (<6.0%, 6.0%–6.5%, ≥6.5%)^22^ and ADA 2020 (<5.7%, 5.7%–6.5%, ≥6.5%)^23^ criteria. Discrimination was evaluated by estimating Harrell’s concordance index (C-index) from Cox proportional-hazards models for (i) AIRE-DM alone, (ii) HbA1c alone, and (iii) the combined AIRE-DM + HbA1c model, with incident T2DM as the outcome. A bootstrap resampling approach (1 000 replicates) was employed to compare model performance, with optimism correction applied using Harrell’s bias method. Continuous NRI quantified the incremental discrimination afforded by the combined model, and likelihood-ratio tests assessed improvements in model fit. Multivariable Cox models adjusted for HbA1c, age, and sex provided hazard ratios (HRs) per AIRE-DM quartile and per SD increase. Kaplan–Meier analyses determined the time to 5% and 10% cumulative incidence of T2DM within each quartile, with differences between curves evaluated by the log-rank test. Statistical analyses were performed with R 4.2.0 statistical package (R Core Team, Vienna, Austria) and Python (version 3.9). See the Supplementary Methods for more details.

### Explainability analysis

To gain insights into the ECG morphologies related to the AIRE-DM predictions, we trained a variational autoencoder (VAE), following a previously described method,^24^ using median ECG beats. The median beats were extracted using the BRAVEHEART software, as outlined in an earlier study.^25^ See Supplementary Methods for more details. The VAE architecture is depicted in Supplementary material online, *Figure S2*.

### Phenome-wide association study

To investigate the associations of the AIRE-DM score, we performed phenome-wide association studies (PheWAS). Using linear regression, the residual of AIRE-DM score was calculated, after adjusting for age, age^2^, and sex. Univariate correlation was then performed using the residual to investigate the association between AIRE-DM scores and 1452 phenotypes in the UKB and echocardiographic parameters in the BIDMC cohort. Bonferroni correction was applied to account for multiple comparisons. See Supplementary Methods for more details.

### Genome-wide association study

To uncover genetic associations with AIRE-DM predictions, we conducted a genome-wide association study (GWAS) using the UKB dataset. See Supplementary Methods for more details.

## Results

In the BIDMC cohort, participants were followed for a mean of 5.47 ± 5.81 (SD) years; during this period, 34 938 (18.4%) subjects died during follow-up and 27 535 (14.5%) developed incident T2DM. In the UKB cohort, the mean follow-up was 3.01 ± 2.66 (SD) years, with 790 (1.2%) deaths and 418 (0.6%) incident T2DM cases. In ELSA-Brasil, the mean follow-up period was 10.4 ± 2.9 years (SD); 599 (4.4%) subjects died during follow up and 1687 (14.5%) developed incident T2DM (*Table 1*).

### Artificial intelligence-enhanced electrocardiography predicts onset of future type 2 diabetes mellitus in individuals without diabetes and is additive to the American Diabetes Association Diabetes Risk Test

**Figure 1:**
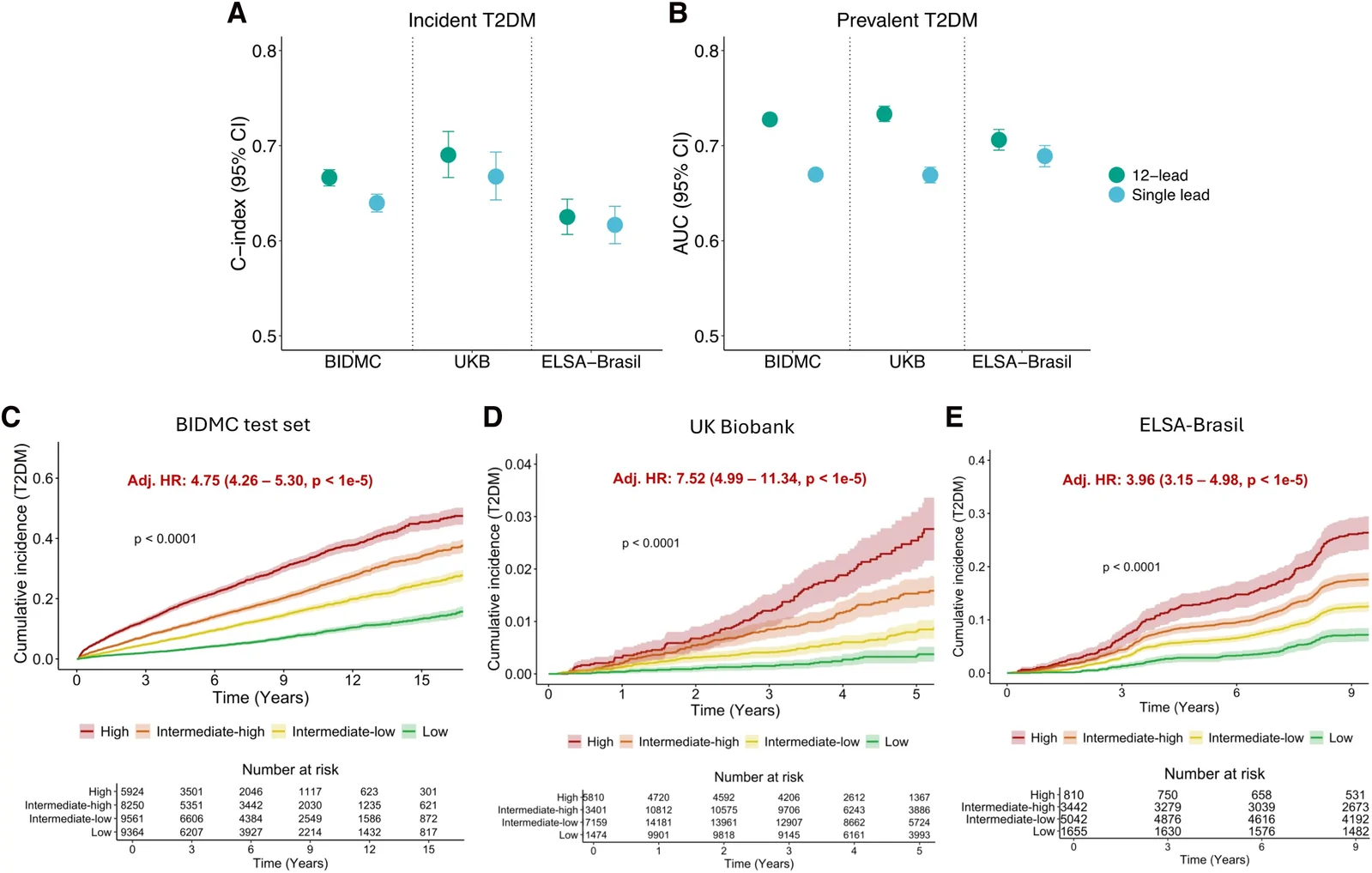
Performance summary of AI-ECG Risk Estimator for Diabetes Mellitus (AIRE-DM) in detecting prevalent type 2 diabetes mellitus (T2DM) and predicting future incident T2DM. Panel (*A*) illustrates AIRE-DM’s performance in predicting incident T2DM in individuals without T2DM, quantified by Harrell’s concordance index (C-index). Panel (*B*) shows its performance in detecting prevalent T2DM, quantified by the area under the receiver operating characteristic curve (AUC). In both panels, results are shown for the 12-lead and single-lead AIRE-DM models. Panels (*C*)–(*E*) display Kaplan–Meier curves for Beth Israel Deaconess Medical Center (BIDMC), UK Biobank, and ELSA-Brasil participants without T2DM, respectively, stratified into quartiles of predicted risk. In all three cohorts, individuals in the highest risk quartile had a significantly higher cumulative incidence of T2DM compared with those in the lowest quartile. Hazard ratios were adjusted for age and sex. The log-rank test was used to derive the *P* value for the differences in cumulative incidence of future T2DM across the risk quartiles.

AI-ECG Risk Estimator for Diabetes Mellitus achieved moderate ability to identify individuals without diabetes who would develop future incident T2DM with a C-index of 0.673 (0.663–0.683) in the BIDMC cohort, 0.689 (0.663–0.715) in the UKB, and 0.625 (0.605–0.643) in ELSA-Brasil (*Figure 1A*). The model was well calibrated in all three cohorts, with Brier Scores of 0.164 (0.162–0.165), 0.145 (0.143–0.146), and 0.119 (0.117–0.120) for BIDMC, UKB, and ELSA-Brasil, respectively (see Supplementary material online, *Figure S1*). A single lead AIRE-DM model demonstrated reasonable performance in identifying individuals at risk of incident T2DM (C-index: BIDMC—0.648 (0.637–0.659); UKB—0.669 (0.666–0.715); ELSA-Brasil—0.625 (0.607–0.644) (*Figure 1A*).

In all three cohorts, AIRE-DM effectively stratified the risk of future T2DM across quartiles (*Figure 1C–E*). Compared with the lowest risk quartile, the highest risk quartile showed age- and sex-adjusted HRs of 4.75 (4.26–5.30) in BIDMC, 7.52 (4.99–11.34) in UKB, and 3.96 (3.15–4.98) in ELSA-Brasil (*Figure 1C–E*). Adjusted HRs for the AIRE-DM continuous score are provided in Supplementary material online, *Table S1*.

For subsets of individuals with available ADA DRT, we then assessed whether AIRE-DM adds incremental value to the ADA DRT (*Table 2*). In the BIDMC cohort, the modified ADA DRT alone achieved a C-index of 0.656 (0.645–0.667). AIRE-DM alone outperformed the modified ADA DRT, achieving a C-index of 0.663 (0.651–0.674) (*P* < 1e-5). Combining AIRE-DM with ADA DRT significantly improved model discrimination, reaching a C-index of 0.686 (0.670–0.702) (*P* < 1e-5), with an NRI of 0.331 (0.271–0.389).

**Table 2:** Comparative performance of AIRE-DM and the ADA DRT in detecting prevalent T2DM and predicting future T2DM in the BIDMC cohort, UK Biobank and ELSA-Brasil

Incident T2DM						
Cohort	Model	C-index (95% CI)	*P* value	NRI	Event NRI	Non-event NRI
BIDMC	AIRE-DM	0.663 (0.651–0.674)		NA	NA	NA
	Modified ADA DRT	0.656 (0.645–0.667)	<1e-5	0.33 (0.27–0.39)	0.15 (0.097–0.19)	0.19 (0.17–0.21)
	Modified ADA DRT + AIRE-DM	0.686 (0.670–0.702)				
UKB	AIRE-DM	0.688 (0.667–0.712)		NA	NA	NA
	ADA DRT	0.717 (0.683–0.752)	<1e-5	0.39 (0.27–0.51)	0.19 (0.091–0.28)	0.20 (0.16–0.24)
	ADA DRT + AIRE-DM	0.747 (0.716–0.777)				
ELSA-Brasil	AIRE-DM	0.623 (0.604–0.643)		NA	NA	NA
	ADA DRT	0.667 (0.650–0.685)	<1e-5	0.21 (0.15–0.29)	0.021 (−0.022–0.078)	0.19 (0.16–0.23)
	ADA DRT + AIRE-DM	0.684 (0.657–0.710)				

Prevalent T2DM						
Cohort	Model	AUC (95% CI)	*P* value	NRI	Event NRI	Non-Event NRI
BIDMC	AIRE-DM	0.724 (0.718–0.730)		NA	NA	NA
	Modified ADA DRT	0.696 (0.690–0.702)	<1e-5	0.48 (0.45–0.51)	0.22 (0.20–0.25)	0.26 (0.24–0.27)
	Modified ADA DRT + AIRE-DM	0.745 (0.739–0.751)				
UKB	AIRE-DM	0.735 (0.725–0.745)		NA	NA	NA
	ADA DRT	0.761 (0.753–0.770)	<1e-5	0.50 (0.46–0.54)	0.22 (0.18–0.26)	0.28 (0.27–0.29)
	ADA DRT + AIRE-DM	0.797 (0.789–0.806)				
ELSA-Brasil	AIRE-DM	0.707 (0.696–0.718)		NA	NA	NA
	ADA DRT	0.762 (0.753–0.772)	<1e-5	0.40 (0.36–0.44)	0.13 (0.098–0.17)	0.27 (0.25–0.29)
	ADA DRT + AIRE-DM	0.787 (0.778–0.797)				

Performance metrics comparing AIRE-DM with the ADA DRT for predicting future T2DM and detecting prevalent T2DM in the BIDMC test set, UK Biobank and ELSA-Brasil. C-index refers to Harrell’s concordance index for time-to-event outcomes; AUC refers to the area under the receiver operating characteristic curve. The continuous net reclassification index (cNRI) is reported alongside its event and non-event components. The table shows that for both incident and prevalent T2DM, the integration of AIRE-DM with the ADA DRT significantly improves predictive accuracy and net reclassification compared with the ADA DRT alone. *P* values were derived from likelihood ratio tests.

ADA DRT, American Diabetes Association Diabetes Risk Test; AIRE-DM, AI-ECG Risk Estimator for Diabetes Mellitus; AUC, area under the receiver operating characteristic curve; CI, confidence interval; C-index, concordance index; NA, not applicable; NRI, net reclassification index; T2DM, type 2 diabetes mellitus.

In the UKB, AIRE-DM alone achieved a C-index of 0.688 (0.667–0.713). American Diabetes Association Diabetes Risk Test achieved a C-index of 0.717 (0.682–0.752), outperforming AIRE-DM (*P* < 1e-5). However, combining AIRE-DM with ADA DRT improved the model discrimination with a C-index of 0.747 (0.716–0.777), *P* < 1e-5, and an NRI = 0.368 (0.248–0.470). In ELSA-Brasil, AIRE-DM alone achieved a C-index of 0.623 (0.604–0.643) and ADA DRT 0.667 (0.650–0.685). Combining AIRE-DM with ADA DRT again significantly improved the model discrimination with a C-index of 0.684 (0.657–0.710), *P* < 1e-5, and an NRI of 0.212 (0.154–0.294).

**Figure 2:**
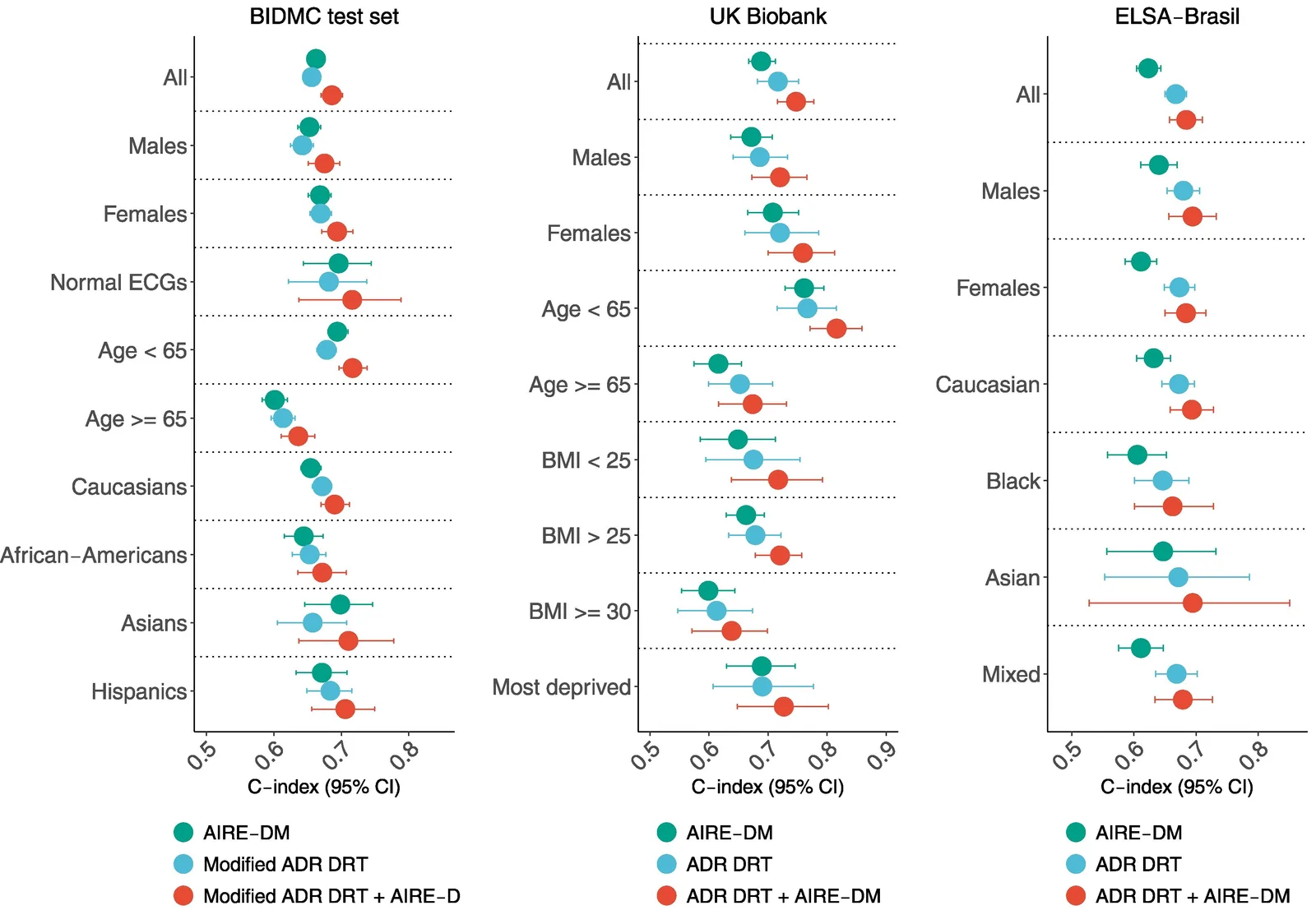
Comparison of predictive performance for incident type 2 diabetes mellitus (T2DM): AI-ECG Risk Estimator for Diabetes Mellitus (AIRE-DM) vs. American Diabetes Association Diabetes Risk Test (ADA DRT) across demographic and ethnic subgroups. Panels (*A*)–(*C*) display Harrell’s concordance index (C-index) (95% confidence interval) for incident T2DM prediction by AIRE-DM (green), ADA DRT (blue), and the combined AIRE-DM and ADA DRT (red) across different demographic and ethnic subgroups in the Beth Israel Deaconess Medical Center (BIDMC) cohort, UK Biobank, and ELSA-Brasil, respectively.

Importantly, the additive nature of AIRE-DM to ADA DRT was reflected across all demographic, ethnic and socioeconomic subgroups in all three cohorts (*Figure 2*). There was also improved performance in subjects under 65 years, those with normal ECGs, and those of Asian ethnicity. Importantly, performance was maintained in individuals of African-American and Hispanic heritage, as well as in subjects with the greatest relative deprivation (defined as the bottom three bottom deciles of Index of Multiple Deprivation).

### AI-ECG Risk Estimator for Diabetes Mellitus detects prevalent type 2 diabetes mellitus and is additive to the American Diabetes Association Diabetes Risk Test

AI-ECG Risk Estimator for Diabetes Mellitus accurately identified existing T2DM in the BIDMC cohort [area under the receiver operating characteristic curve (AUC) 0.724 (0.718–0.730)]. In comparison, the modified ADA DRT achieved an AUC of 0.696 (0.690–0.702). Incorporating AIRE-DM with the modified ADA DRT yielded an improved detection of prevalent T2DM [AUC: 0.745 (0.739–0.751), *P* < 1e-5, continuous NRI = 0.48 (0.45–0.451)] (*Table 2*). AIRE-DM performed similarly in the UKB cohort, achieving an AUC of 0.735 (0.725–0.745). The ADA DRT model achieved an AUC of 0.761 (0.753–0.770). Notably, combining AIRE-DM with ADA DRT achieved a significant improvement in performance over ADA DRT alone (*Table 2*) [AUC: 0.797 (0.789–0.806), NRI = 0.50 (0.46–0.54)]. In ELSA-Brasil, AIRE-DM achieved an AUC of 0.706 (0.695–0.717). The ADA DRT achieved an AUC of 0.762 (0.753–0.772). Combining AIRE-DM with ADA DRT led to a significant improvement in model discrimination, with an AUC of 0.787 (0.778–0.797) and an NRI of 0.40 (0.36–0.44). Additionally, a single lead AIRE-DM again demonstrated reasonable accuracy in detecting prevalent T2DM [AUC: BIDMC—0.670 (0.664–0.676); UKB—0.669 (0.661–0.677); ELSA-Brasil—0.689 (0.678–0.700)] (*Figure 1B*).

### AI-ECG Risk Estimator for Diabetes Mellitus enhances prediction of future type 2 diabetes mellitus when combined with glycated haemoglobin

**Figure 3:**
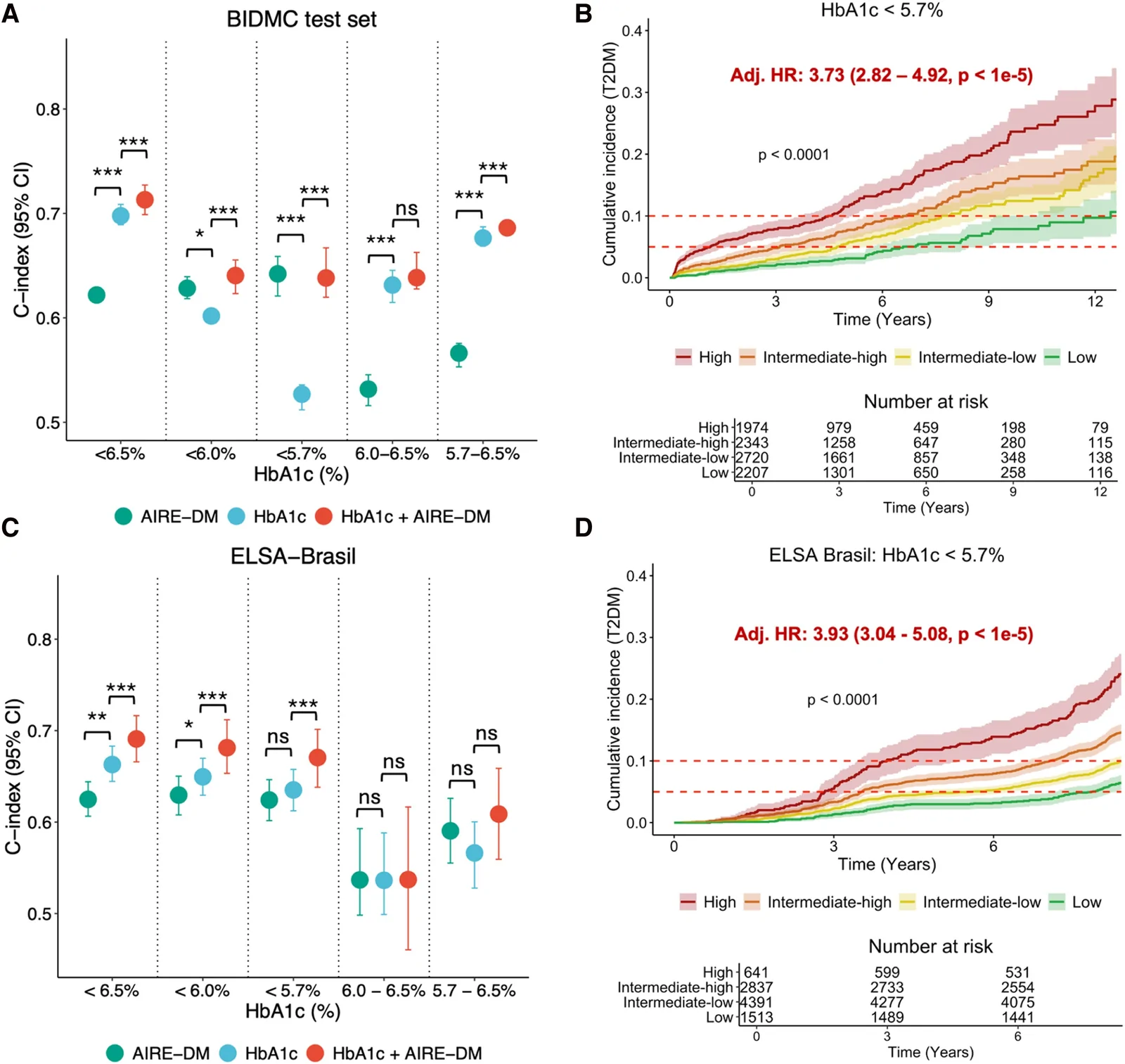
Comparison of predictive performance of AI-ECG Risk Estimator for Diabetes Mellitus (AIRE-DM) and glycated haemoglobin (HbA1c) for incident type 2 diabetes mellitus (T2DM) across glycaemic strata and cumulative incidence by risk quartile. Panels (*A*) and (*C*) display Harrell’s concordance index (C-index) (95% confidence interval) for incident T2DM prediction by AIRE-DM alone (green), HbA1c alone (blue), and the combined model (red) across standard HbA1c categories in the Beth Israel Deaconess Medical Center (BIDMC) test set (*A*) and the ELSA-Brasil cohort (*C*). Nested Cox models were compared by likelihood-ratio test, and non-nested models by partial likelihood-ratio test. Panels (*B*) and (*D*) show Kaplan–Meier curves for participants with baseline HbA1c < 5.7%, stratified by AIRE-DM risk quartile in BIDMC (*B*) and ELSA-Brasil (*D*), adjusted for continuous HbA1c, age, and sex. Differences between risk quartiles were evaluated by the log-rank test.

Furthermore, in a subset of BIDMC patients with available HbA1c data, we evaluated the added predictive value of AIRE-DM to HbA1c at various thresholds for predicting future incident T2DM (*Figure 3A*). The greatest additive value of AIRE-DM was in the normal range for HbA1c, <5.7% (*n* = 9244 individuals). Here, AIRE-DM was superior to HbA1c, achieving a C-index of 0.642 (0.621–0.659), while HbA1c alone provided little predictive value, with a C-index of 0.527 (0.512–0.536) (*P* < 1e-5). Adding HbA1c to AIRE-DM yielded no improvement, with a C-index of 0.638 (0.620–0.667), NRI of −0.111 (−0.154–0.214), *P* = 0.960. The highest-risk AIRE-DM quartile had significantly higher risk of future T2DM vs. the lowest-risk quartile [HR = 3.73 (2.82–4.92, *P* < 1e-5)] after adjustment for HbA1c, age, and sex. Furthermore, the time required for the cumulative incidence of T2DM to reach 5% and 10% increased progressively across quartiles—from 1.15 and 4.46 years in the highest-risk quartile to 6.58 and 12.4 years in the lowest-risk quartile (*Figure 3B*).

In a subset of ELSA-Brasil participants with available HbA1c data at baseline (*n* = 13 717), AIRE-DM was significantly additive to HbA1c in all HbA1c categories except for 6.0%–6.5% and 5.7%–6.5% (*Figure 3C*). In participants with normoglycaemia (HbA1c < 5.7%, *n* = 9382), AIRE-DM alone was non-inferior to HbA1c, achieving a C-index of 0.624 (0.602–0.647), compared with HbA1c (C-index of 0.635 (0.612–0.658), *P* = 0.169). Adding AIRE-DM to HbA1c significantly improved the C-index to 0.671 (0.638–0.658) (*P* < 1e-5), NRI = 0.317 (0.222–0.407). Time to 5% cumulative incidence ranged from 2.71 years in the highest-risk quartile to 7.49 years in the lowest-risk quartile. The 10% cumulative incidence was reached at 3.64 years in the highest-risk quartile but was not attained by the lowest-risk quartile during the follow-up period.

### AI-ECG Risk Estimator for Diabetes Mellitus predicts diabetes-related adverse events

**Figure 4:**
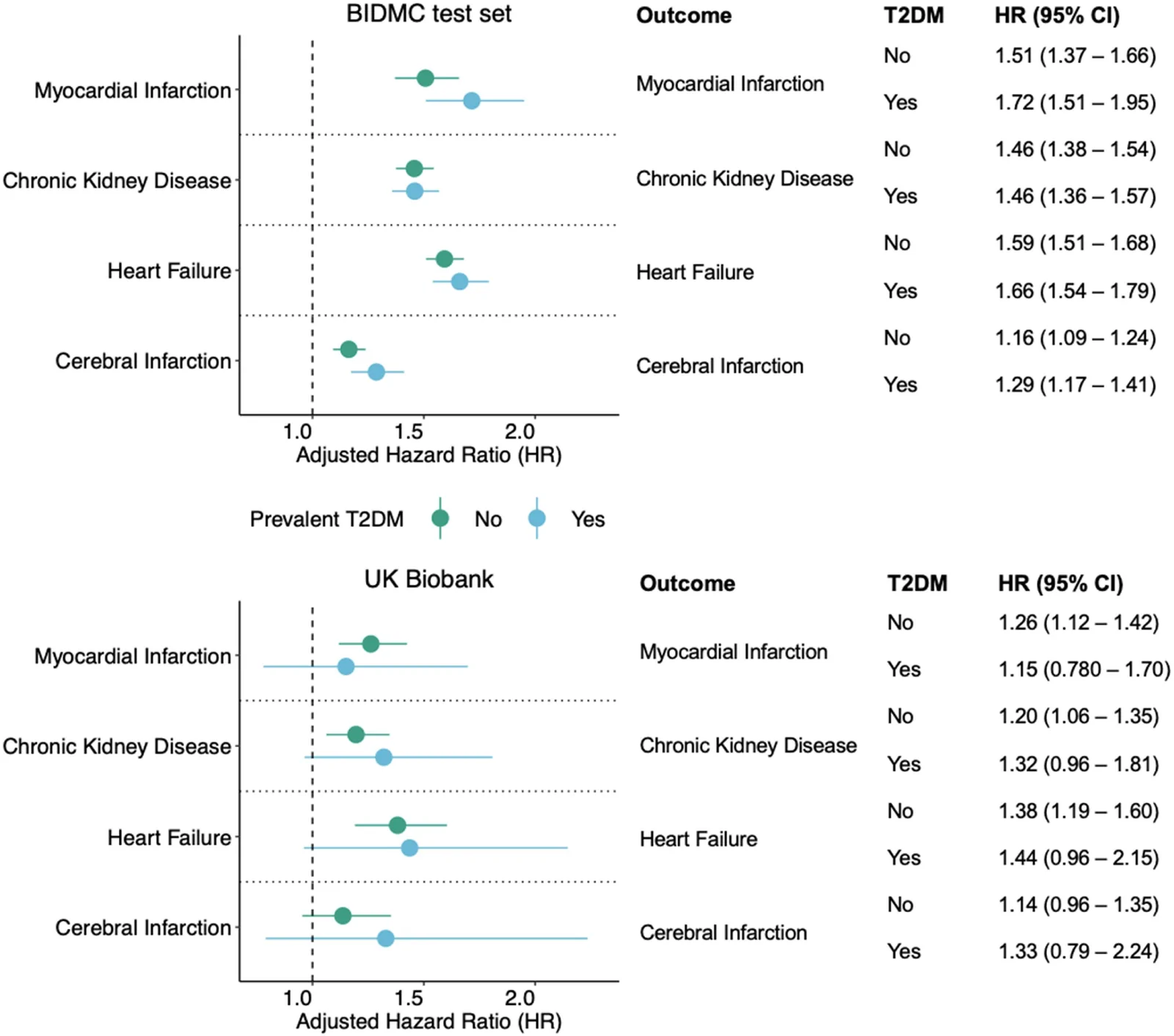
AI-ECG Risk Estimator for Diabetes Mellitus (AIRE-DM) is independently associated with type 2 diabetes mellitus (T2DM)-related adverse outcomes. In adjusted Cox models, the AIRE-DM score independently predicts T2DM-related adverse outcomes in subjects from the Beth Israel Deaconess Medical Center (BIDMC) cohort (*A*), regardless of whether they have prevalent T2DM. For the UK Biobank (UKB) cohort (*B*), the score is predictive for individuals without prevalent T2DM, but not significant for those with prevalent T2DM. Covariates for BIDMC analysis included age, sex, systolic and diastolic blood pressures, smoking status, prevalent hypertension, hyperlipidaemia, and ethnicity. UKB analyses additionally included body mass index as a covariate. Hazard ratio refers to one standard deviation of AIRE-DM score.

To further explore AIRE-DM’s utility, we examined its ability to predict diabetes-related future adverse events. In BIDMC, AIRE-DM score was an independent predictor of myocardial infarction (MI), heart failure (HF), chronic kidney disease (CKD), and ischaemic stroke, regardless of the status of prevalent T2DM (*Figure 4*). In the UKB, AIRE-DM was also an independent predictor of MI, HF, and CKD, but not stroke, in subjects without prevalent T2DM. For subjects with T2DM, AIRE-DM showed a consistent but non-significant trend towards predicting these events (*Figure 4*).

### AI-ECG Risk Estimator for Diabetes Mellitus score is explainable and biologically plausible

**Figure 5:**
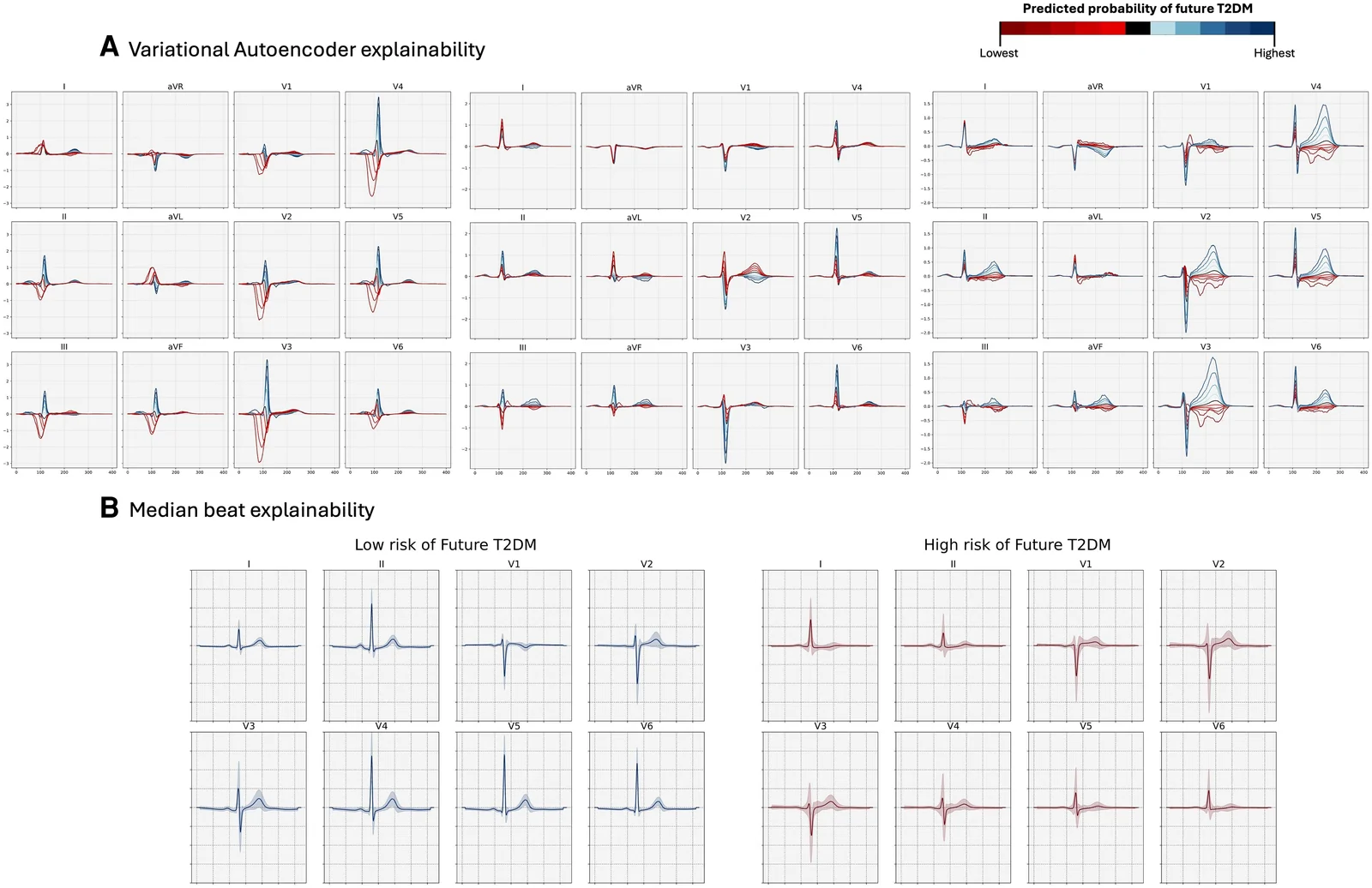
Explainability analysis. Two explainability approaches were applied to explore the electrocardiogram (ECG) morphologies associated with AI-ECG Risk Estimator for Diabetes Mellitus (AIRE-DM) predictions. Variational autoencoder-derived latent features were analysed using linear regression to predict AIRE-DM outputs, with the three most significant features shown, highlighting abnormalities in QRS morphology and T wave morphology, including T wave inversion and biphasic T waves, as being most associated with high diabetes mellitus risk. Additionally, median ECG waveforms (mean ± SD, shaded region) were generated for the 10 000 highest and lowest predicted survival ECGs from the Beth Israel Deaconess Medical Center test set, identifying poor precordial R wave progression as the feature most strongly associated with a high risk of future type 2 diabetes mellitus (T2DM).

**Figure 6:**
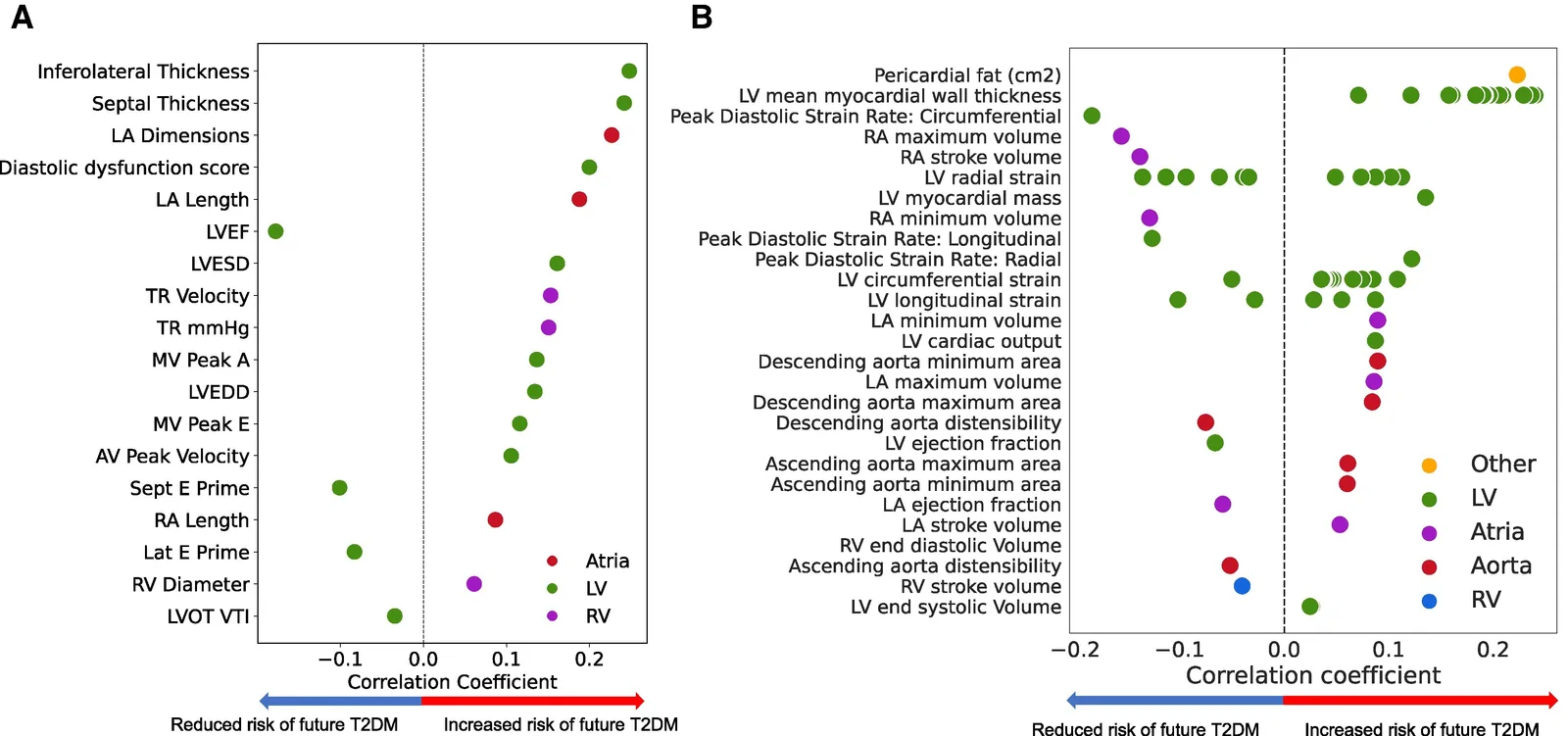
Phenotypic associations of AI-ECG Risk Estimator for Diabetes Mellitus (AIRE-DM) predictions. Panel (*A*) illustrates the associations between AIRE-DM risk predictions and echocardiographic parameters in Beth Israel Deaconess Medical Center test set and panel (*B*) depicts the associations with cardiovascular magnetic imaging parameters in the UK Biobank. LA, left atrium; LV, left ventricle; LVEDD, LV end-diastolic diameter; LVEF, LV ejection fraction; LVESD, LV end-systolic diameter; LVOT, LV outflow tract; MV, mitral valve; TR, tricuspid regurgitation.

**Figure 7:**
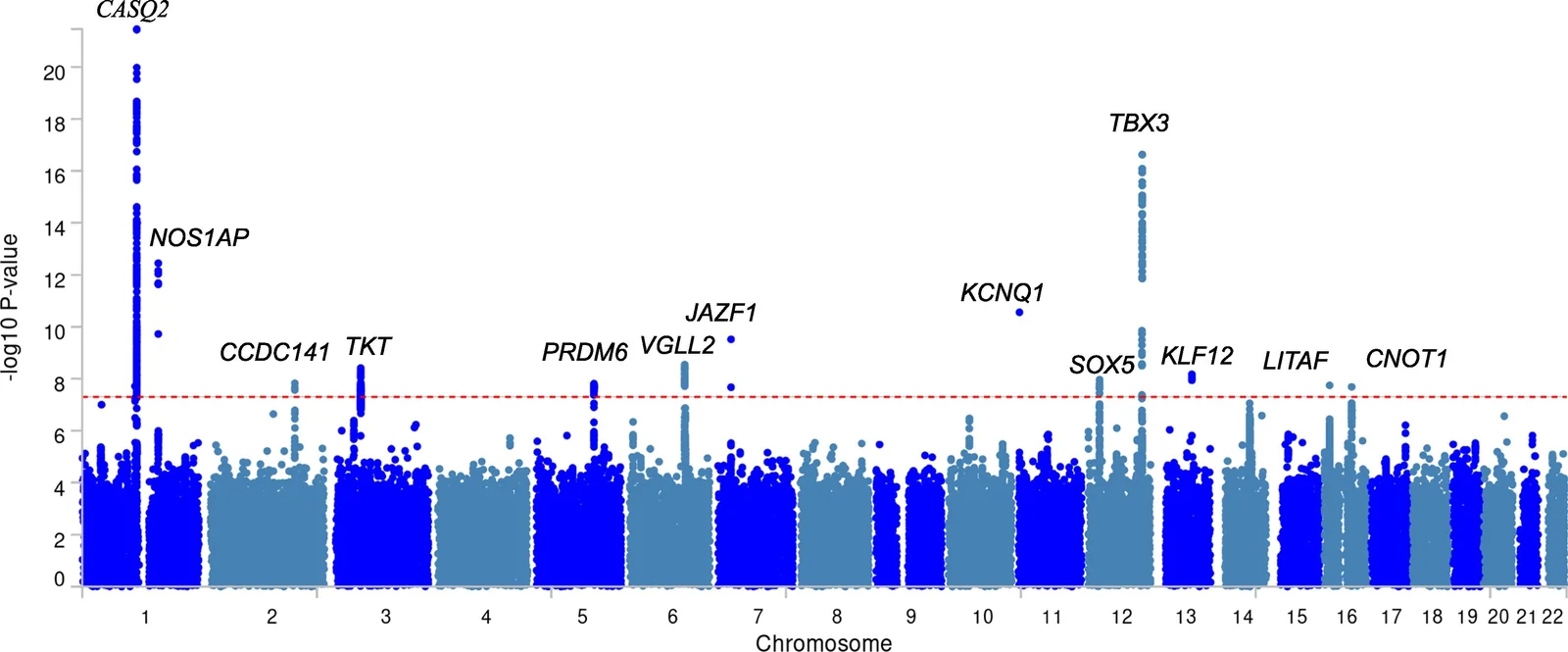
Genotypic associations of AI-ECG Risk Estimator for Diabetes Mellitus (AIRE-DM) predictions. A Manhattan plot of genomic loci associated with AIRE-DM scores. Nearest genes to significant single nucleotide polymorphisms are shown. The red line depicts the genome-wide significant threshold (*P* value < 5e-8).

To better understand why AIRE-DM is effective, we next examined its explainability and biological plausibility. First, a VAE and median ECG waveform analysis revealed that key electrocardiographic features—longer QRS duration, LBBB QRS morphology, abnormal T wave morphology, and poor precordial R wave progression—were strongly associated with higher predicted risk of future T2DM (*Figure 5*). Second, using PheWAS in the UKB, we identified significant cardiac and non-cardiac phenotypes associated with AIRE-DM, including triglycerides, HbA1c, systolic and diastolic blood pressure, and cardiac index. Significant positive echocardiographic (BIDMC) and cardiovascular magnetic imaging (CMR) associations (UKB) included pericardial fat, left ventricular wall thickness and mass, diastolic dysfunction score, and radial peak diastolic strain rate. Significant negative associations included left ventricular ejection fraction, circumferential and longitudinal peak diastolic strain rates, and right atrial volumetric parameters (*Figure 6A* and *B*). Finally, a GWAS of AIRE-DM score in the UKB (*Figure 7*), identified variants adjacent to *CASQ2*, *TBX3*, *NOS1AP*, *TKT*, *VGLL2*, and *PRDM6*. A total of 10.5% (7.9%–13.0%) of the total phenotypic variance was explained by genetic variance. The QQ plots are shown in Supplementary material online, *Figure S3*.

## Discussion

We introduce AIRE-DM, the first robust AI-ECG model designed for prediction of future T2DM in individuals. Developed and internally validated in a secondary care cohort of over 1.1 million ECGs and externally validated in the UKB healthy volunteer cohort and ELSA-Brasil civil servants, AIRE-DM demonstrates consistent performance across various demographic groups, including sex and ethnicity. We show that integrating AIRE-DM with traditional risk scores, such as ADA DRT, significantly improves prediction of future T2DM, indicating its potential value for T2DM prediction and risk stratification. Most importantly, our model has utility in predicting future T2DM in patients who have not yet developed pre-diabetes (i.e. HbA1c is in the normal range). In addition, our deep exploratory analyses uncovered biologically plausible associations with AIRE-DM predictions. These findings demonstrate that AIRE-DM could offer a glucose-independent screening modality capable of identifying individuals at elevated risk for T2DM prior to the onset of overt dysglycaemia. If implemented early, it may have the potential to delay diagnosis or even prevent the onset of microvascular and macrovascular complications that are thought to begin during the preclinical phase of the disease.

### AI-ECG Risk Estimator for Diabetes Mellitus predicts future type 2 diabetes mellitus and enhances prediction when combined with routine risk factors and risk scores

Previous studies primarily focused on using AI-ECG for the detection of prevalent T2DM only.^26,27^ The unique feature of AIRE-DM is its ability to predict the risk of future T2DM in individuals without overt dysglycaemia. Its predictive ability was validated internally within the BIDMC and externally in a population-based volunteer cohort, the UKB, showcasing consistent performance across diverse demographic and ethnic groups. AIRE-DM effectively stratified risk across cohorts of differing baseline incidence. Notably, AIRE-DM was non-inferior to the ADA DRT in the BIDMC cohort, and the addition of AIRE-DM to the ADA DRT in both cohorts significantly improved predictive accuracy and reclassification, suggesting that AIRE-DM captures unique risk-related information about future T2DM not accounted for by the ADA DRT. These findings highlight the potential of AIRE-DM to enhance risk stratification and guide preventive interventions in individuals at high risk of developing T2DM. The single-lead ECG version of AIRE-DM also retained most of its predictive performance for prediction of future T2DM, again underscoring its potential for integration into wearable devices.

### Enhancing undiagnosed type 2 diabetes mellitus detection with AIRE-DM

AIRE-DM demonstrates promising performance for opportunistic detection of individuals with undiagnosed T2DM in both secondary care and population-based cohorts. Importantly, AIRE-DM is additive to the ADA DRT,^21^ designed to identify those at risk of undiagnosed diabetes to guide screening decisions. Notably, a single-lead ECG version of AIRE-DM demonstrated reasonable accuracy in detecting prevalent T2DM, highlighting its potential utility in wearable technology for widespread screening and early detection of undiagnosed T2DM. Therefore, this low-cost, non-invasive test could serve as a valuable screening tool to identify individuals who would benefit from further diagnostic tests to detect currently undiagnosed diabetes, potentially improving patient outcomes and reducing the burden of undiagnosed T2DM. Whilst pathways for the diagnosis of T2DM are well-established in high-income countries using HbA1C testing, AIRE-DM could have utility in other resource settings, where it could signal the need for diabetes testing in patients presenting with cardiac disease.

### AI-ECG Risk Estimator for Diabetes Mellitus risk stratification in normoglycaemic individuals

Glycated haemoglobin has previously been shown to be insensitive at identifying individuals with early impairment in β-cell function^28,29^ and to have limited sensitivity for detecting pre-diabetes.^30^ Furthermore, current glucose-centric screening may detect T2DM only after overt metabolic derangement, thereby potentially delaying preventive measures in higher risk individuals.^5^ Accordingly, AIRE-DM was evaluated in two distinct normoglycaemic (HbA1c < 5.7%) cohorts by comparing AIRE-DM with HbA1c. In the BIDMC cohort, AIRE-DM identified individuals at risk of future T2DM despite the minimal prognostic value of HbA1c alone. In ELSA-Brasil, AIRE-DM demonstrated non-inferior predictive discrimination compared with HbA1c and, when combined with HbA1c, further enhanced predictive performance.

Overall, these findings confer two principal advantages of AIRE-DM over HbA1c in individuals with normoglycaemia. First, AIRE-DM may detect early insulin resistance by capturing subclinical cardiac structural and electrophysiological changes not reflected in traditional glycaemic markers. Second, it identifies high-risk individuals who would—but current screening methods—be defined as ‘normal’, enabling earlier preventive interventions to delay or prevent the onset of T2DM and its complications, in line with population-level prevention principles. The time-to-5% and time-to-10% cumulative incidence analyses in this normoglycaemic subgroup are the most clinically interpretable demonstration of AIRE-DM’s added value, providing a concrete measure of the lead time afforded by the model over existing screening.

Consistent with this, AIRE-DM was also independently associated with future adverse cardiovascular events in non-diabetic individuals across both cohorts in adjusted analyses, supporting its broader role as a marker of cardiometabolic risk that extends beyond glucose-centric definitions. These associations are likely to reflect AIRE-DM’s capture of broader preclinical cardiometabolic risk.

### Other non-glycaemic based artificial intelligence type 2 diabetes mellitus detection methods

While methods like clinical metadata analysis,^31^ facial texture features,^32^ retinal fundus imaging,^33^ photoplethysmography,^34^ and external eye photographs^35^ have shown higher predictive performance (C-indices > 0.7) for detecting or predicting T2DM, they often require specialized equipment, controlled environments, and trained personnel. These limitations hinder their widespread applicability in routine outpatient or primary care settings. In contrast, ECGs are widely performed across healthcare environments, with millions conducted daily worldwide. AI-ECG models like AIRE-DM are highly relevant for opportunistic screening and risk prediction due to the ease of accessing ECG signals. The integration of ECG-based detection into existing clinical workflows without additional resources highlights the practicality and accessibility of AI-ECG as a valuable tool for early detection and intervention strategies for T2DM. However, directly comparing performances of models of different modalities across entirely different populations is challenging. Differences in study design, patient demographics, and underlying data characteristics make it difficult to draw definitive conclusions about one modality’s superiority over another. Therefore, while the higher C-indices of some alternative methods are notable, they should not be taken as a straightforward benchmark against which to measure AI-ECG models like AIRE-DM.

### Biological associations and interpretability of AI-ECG Risk Estimator for Diabetes Mellitus in predicting future type 2 diabetes mellitus

Our in-depth analyses using PheWAS, GWAS, and explainability techniques revealed associations between AIRE-DM predictions and cardiac traits (e.g. abnormal blood pressure, cardiac function, and morphology) as well as metabolic traits (e.g. triglycerides, HbA1C, and visceral adipose tissue). Electrocardiogram explainability highlighted associations with QRS and T wave abnormalities, consistent with evidence linking prolonged QRS duration and abnormal repolarization to diabetes and pre-diabetes.^36,37^

Echocardiographic and CMR parameters associated with AIRE-DM included increased left ventricular wall thickness, diastolic dysfunction, atrial enlargement, and increased pericardial fat. Notably, interventricular septal and posterior wall thicknesses are elevated in patients with T2DM, even in the absence of hypertension,^38^ and progressively increase from normoglycaemic to diabetic states.^39^ Diastolic dysfunction emerges as one of the earliest cardiac changes in pre-diabetes, likely due to insulin resistance,^40,41^ and elevated epicardial adipose tissue is observed in both pre-diabetic and diabetic states.^42^ However, as we did not adjust the PheWAS analyses for BMI, we cannot fully adjudicate whether these associations are independent of obesity.

Genome-wide association study identified loci adjacent to known regulators of cardiac morphology (e.g. *CASQ2, TBX3, PRDM6*), metabolic traits (e.g. *NOS1AP, TKT, PRDM6*), and arterial stiffness (e.g. *VGLL2, PRDM6*). These genes are implicated in pathways regulating myocardial structure and function,^43–47^ glycaemic control,^48–50^ and arterial stiffness,^51–53^ aligning with mechanisms underlying the pathogenesis of T2DM.

### Clinical implications

Using routinely collected ECGs, AIRE-DM has potential as a non-invasive, cost-effective tool for opportunistic screening of individuals at high risk of existing or future T2DM. While the moderate predictive performance may limit standalone use in clinical settings, AIRE-DM’s ability to complement existing risk scores or serve as an initial screening tool demonstrates practical value.

There are four potential applications for AIRE-DM. First, in low- and middle-income countries (LMICs), where laboratory-based screening is often limited and resources are scarce, AIRE-DM could serve as an opportunistic screening tool—flagging individuals who should then be tested for T2DM. This would be particularly relevant in settings where ECG infrastructure already exists in remote primary care, such as the Telehealth Network of Minas Gerais, which has performed over 3.8 million tele-ECGs across 813 municipalities in Minas Gerais.^54^ However, any deployment would be contingent on existing diagnostic and management pathways, given that the cascade of diabetes care in LMICs is often constrained at multiple stages.^55^ Second, although its deployment in routine clinical pathways to identify undiagnosed T2DM may be of lower added value (given the widespread use of HbA1c), it could still augment traditional methods. Third, and most importantly, AIRE-DM could be used in a precision medicine approach to identify individuals at high risk of future T2DM—independent of the conventional, HbA1c-anchored definitions—thereby enabling earlier intervention to prevent disease progression. For example, patients undergoing ECG in any clinical context, identified as being at high risk for existing or future T2DM, could be targeted for biochemical confirmation followed by regular surveillance, intensive lifestyle modification, and consideration of pre-emptive pharmacotherapy where indicated.^56–59^ Fourth, single-lead models could be deployed on wearable devices to further enhance screening.

In all four applications, the use of AIRE-DM will need to be prospectively evaluated against existing screening pathways for T2DM, such as ADA DRT and risk factor assessment,^60^ with a focus on its impact on clinical decision-making and patient outcomes as part of a broader risk stratification strategy.

The use of AIRE-DM could be particularly effective in groups of patients with higher rates of progression from pre-diabetes to diabetes, such as South Asian individuals,^61–63^ older Black adults,^64^ and individuals from deprived backgrounds.^62^ In these groups, the window for effective prevention may be shorter once dysglycaemia becomes established. We have demonstrated that AIRE-DM consistently and significantly improves the prediction of future T2DM, when combined with common clinical risk factors, across various demographic groups, including in African-Americans, Asians, and Hispanic/Latino patients, suggesting that it may have potential to supersede traditional risk models that perform variably in different ancestry groups. Importantly, AIRE-DM maintains its performance in the most deprived patients, a group often disproportionately affected by algorithmic bias.^65^

The clinical impact of AIRE-DM will vary by deployment setting. The cumulative 5-year incidence of T2DM in the highest AIRE-DM quartile ranged from ∼3% in the UKB to nearly 20% in ELSA-Brasil, reflecting substantially different baseline event rates between a population-based volunteer cohort and a longitudinal cohort of civil servants with higher cardiometabolic risk. The number of individuals needed to screen to identify one future case will, therefore, depend on the population in which AIRE-DM is deployed. AIRE-DM is, however, highly scalable: it operates on ECGs already acquired for other clinical indications, keeping the marginal cost per screen low even in lower-incidence populations. Furthermore, it runs on the standard digital ECG signal as input and requires no additional hardware beyond existing ECG infrastructure.

### Limitations

Several limitations of our study need to be acknowledged. As with other epidemiological studies, its accuracy relies on the correct coding of T2DM diagnoses, a task complicated by the often-asymptomatic nature of T2DM. This raises the possibility that some patients with developing incident T2DM subsequent to their ECG could have had undiagnosed diabetes at the time their ECG was recorded. The moderate performance of AIRE-DM reflects the inherent challenge of detecting subtle preclinical signs of T2DM in ECGs. While training the model with a larger dataset matched to HbA1c could potentially enhance performance compared with using ICD codes, this approach is inherently limited by intrinsic selection bias, as individuals with available HbA1c measurements are often preselected based on suspicion of pre-diabetes or diabetes. Furthermore, replication of the GWAS findings in independent cohorts is necessary to validate the observed genetic associations. In addition, the BIDMC cohort comprises individuals who underwent ECG for clinical indications, who may differ from those not undergoing ECG. In the UKB and ELSA-Brasil, however, ECGs were undertaken as protocolized research procedures, mitigating this concern.

## Conclusion

In conclusion, we present the first robustly evaluated AI-ECG model that can predict future T2DM risk in non-diabetic populations, with validation undertaken in both primary and secondary care cohorts. AIRE-DM enhances T2DM risk prediction and stratification when integrated with traditional risk factors and risk scores and is underpinned by the identification of plausible biological pathways. Uniquely, it has greatest added value in individuals below the pre-diabetic range (HbA1c < 5.7), who would not otherwise be identified as high-risk. Its application as a non-invasive, widespread, and opportunistic screening tool holds promise for early identification of individuals at higher risk of future T2DM beyond traditional risk factor models, enabling timely interventions and personalized management.

## Supplementary Material

ztag118_Supplementary_Data

## Data Availability

UK Biobank data are available upon application (http://www.ukbiobank.ac.uk/). The study was conducted under application numbers 48666 and 47602. The BIDMC dataset is restricted due to ethical limitations. Access to the ELSA-Brasil cohort is available upon request from the study steering committee. Researchers from educational or research institutions may submit requests to obtain the dataset. Such requests should be directed to the corresponding author of the paper. They will then be forwarded and evaluated individually by both the Telehealth Network of Minas Gerais and the ELSA-Brasil Steering Committee.

## Code availability

The code underlying the AI-ECG architecture and discrete-time survival loss functions are available here: https://github.com/antonior92/automatic-ecg-diagnosis and https://github.com/MGensheimer/nnet-survival.
