# Supplementary material for: Artificial intelligence-enhanced electrocardiography for the prediction of future type 2 diabetes mellitus: a model-development and multicentre validation study
Source: Eur Heart J Digit Health. 2026 Jul 22;7(7):ztag118. doi: 10.1093/ehjdh/ztag118 (PMC13525370; doi:10.1093/ehjdh/ztag118)
Supplement: Supplementary file 1 [file ztag118_supplementary_data.docx]

**Artificial Intelligence-Enhanced Electrocardiography for the Prediction of Future Type 2 Diabetes Mellitus: a model-development and multicentre validation study**

**Supplementary material**

Table of Contents

[Supplementary methods	3](#_Toc200965770)

[Ethical approvals	3](#_Toc200965771)

[Cohorts	3](#_Toc200965772)

[ECG pre-processing	4](#_Toc200965773)

[Outcome definition	4](#_Toc200965774)

[Model development	4](#_Toc200965775)

[Subgroup definitions	4](#_Toc200965776)

[Statistical analysis	5](#_Toc200965777)

[AIRE-DM association with HbA1c	5](#_Toc200965778)

[Explainability analysis	5](#_Toc200965779)

[Phenome-wide association study	6](#_Toc200965780)

[Genome-wide association study	6](#_Toc200965781)

[Supplementary Tables/Figures	8](#_Toc200965782)

[Supplementary Table 1: Hazard ratios of Cox regression analyses of AIRE-DM score	8](#_Toc200965783)

[Supplementary Figure 1: Calibration of AIRE-DM for the prediction of future incident T2DM in the BIDMC, UKB and ELSA-Brasil cohorts	9](#_Toc200965784)

[9](#_Toc200965785)

[Supplementary Figure 2: The VAE convolutional architecture	10](#_Toc200965786)

[Supplementary Figure 3: GWAS QQ plots	11](#_Toc200965787)

[References	12](#_Toc200965788)

# Supplementary methods

## Ethical approvals

Ethical clearance for the Beth Israel Deaconess Medical Center (BIDMC) cohort was granted by the Beth Israel Deaconess Medical Center Committee on Clinical Investigations, under IRB protocol # 2023P000042.

Approval for the UK Biobank's research activities as a Research Tissue Bank was obtained from the North West Multi-Centre Research Ethics Committee, with application IDs 48666 and 47602.

The Longitudinal Study of Adult Health (ELSA-Brasil) was approved by the Research

Ethics Committees of the participating institutions and by the National Committee for

Research Ethics (CONEP 976/2006) of the Ministry of Health.

## Cohorts

We examined three cohorts. The Beth Israel Deaconess Medical Center (BIDMC) cohort, a secondary care dataset, consists of routinely gathered data from Beth Israel Deaconess Medical Center in Boston, USA, and comprises individuals aged 16 and above who underwent valid ECGs between 2014 and 2023. Prior ECGs dating back to 2000 were also considered for these participants. Disease status was determined utilising diagnostic International Classification of Diseases (ICD) codes. Subjects were either censored at the time of outcome manifestation or their last in-person hospital interaction.

The UK Biobank (UKB) is a longitudinal study involving over 500,000 volunteers aged 40-69 at enrolment between 2006 and 2010 (1). During the baseline assessment, participants completed health and lifestyle questionnaires, underwent physical measurements (such as height, weight, and blood pressure), and provided samples of blood, urine, and saliva. A subset of participants returned for follow-up visits, which included additional investigations like cardiac magnetic resonance imaging (MRI), brain MRI, and digital ECGs. For our analysis, data from 65,610 subjects with 69,859 digital ECGs taken at the second instance were used. There is evidence of healthy volunteer selection bias (2). Outcome data were linked to cancer and death registries, hospital admissions, and primary care records. Detailed phenotyping using cardiac MRI data has been previously documented (3, 4).

ELSA-Brasil is a cohort study of 15,105 Brazilian public servants, aged 35 to 74 at

enrolment. All active or retired employees of six participating institutions were eligible

for the study.

## ECG pre-processing

The 12-lead ECGs from both cohorts were pre-processed using a bandpass filter at 0.5 to 100 Hz, a notch filter at 60 Hz, and re-sampled to 400 Hz. Zero padding was used to achieve a signal with 4096 samples for each lead for a 10s recording. The quality of the ECG recordings was assessed using the BRAVEHEART software and signals with excessive artefact excluded (5).

## Outcome definition

In the BIDMC, T2DM was defined using the first instance of the following ICD-9/10 codes: 250.00, 250.02, 250.9, 250.92, 250.1, 250.12, 250.2, 250.22, 250.3, 250.32, 250.40, 250.50, 250.52, 250.60, 250.70, 250.72, 250.80, 250.82, 250.90, 250.92, E11, E13, E14, and G59.0.

## Model development

For prediction of future T2DM, the discrete-time interval representing 5 years after the ECG recording was utilised. In contrast, for classification of prevalent T2DM, the initial discrete time interval, corresponding to the time of the ECG recording itself, was employed. Model development and testing were performed with Python (version 3.9) and tensorflow (version 2.9.0).

## Subgroup definitions

We assessed AIRE-DM's performance on a subset of ECGs from the BIDMC dataset, classified as normal by cardiologists. Normal ECGs were identified by searching for "normal ecg" in the reports, excluding those with "abnormal ecg" or "otherwise", and filtering ECGs for heart rate of 60-100 bpm, PR interval <200 ms, QTc interval <470 ms, and QRS duration <120 ms. The “No CVD disease” subgroup defined as individuals with no prevalent or future (within 5 years of each ECG) atherosclerotic cardiovascular disease, HF or ischaemic stroke. The most deprived subgroup was defined as the bottom three Index of Multiple Deprivation (IMD) deciles.

## Statistical analysis

To evaluate AIRE-DM's performance in predicting future T2DM in those classified as non-diabetic, Cox proportional hazard models were employed to assess the association between AIRE-DM predictions and time to T2DM onset. In both cohorts, the hazard ratios were adjusted for age and sex. The AIRE-DM risk score quartiles were defined using the distributions in the BIDMC validation dataset. Kaplan-Meier curves were generated to visualise the cumulative probabilities of T2DM, and log-rank tests were conducted to assess the statistical significance. Calibration of AIRE-DM for the prediction of future T2DM in the BIDMC and UKB cohorts was performed using the R pmcalibration package (version 0.1.0) and Brier Score was calculated using the DescTools package (version 0.99.54).

Statistical analyses were performed using the R 4.2.0 statistical package (R Core Team, Vienna, Austria) and relevant packages such as survival and rms. All tests were two-sided, and p-values < 0.05 were considered statistically significant.

## AIRE-DM association with HbA1c

We then explored the relationship between the AIRE-DM predictions for future T2DM and HbA1c levels at the time of the ECG recording. For the subset of individuals with HbA1c levels available within 90 days of each ECG in the BIDMC cohort, we categorised HbA1c into three groups according to 1) World Health Organization (WHO) (<6.0%, 6.0% - 6.5%, ≥ 6.5%) (8) and 2) American Diabetes Association (ADA) (<5.7%, 5.7% - 6.5%, ≥ 6.5%) (9).

## Explainability analysis

The VAE was constructed using a convolutional encoder/decoder architecture, drawing inspiration from architectures that have been previously employed for ECG analysis (10, 11). In particular, the encoder consisted of six convolutional blocks for feature extraction, tailored specifically for the median beat ECG signal. The decoder architecture was designed to be a symmetrically inverse network of the encoder. The encoder and decoder comprised a total of 1,533,888 and 1,283,976 parameters, respectively. A comprehensive illustration of the networks' architecture is presented in **Supplementary Figure 4**.

## Phenome-wide association study

The UKB dataset includes 1,452 phenotypes consisting of patient measurements, surveys and investigations, including cardiac MRI. Additionally, this approach was applied to subjects in the BIDMC holdout set who had echocardiograms within 60 days of their ECGs, to investigate the associations between the AIRE-DM score and echocardiographic parameters, adjusted for age, age^2^, sex. Bonferroni correction was applied to account for multiple comparisons. The diastolic dysfunction score was determined by the sum of the following criteria: 1) Average E/e' > 14, 2) Septal e' velocity < 7 cm/s or Lateral e' velocity < 10 cm/s, 3) TR velocity > 2.8 m/s, 4) LA volume index > 34 ml/m² (13).

## Genome-wide association study

The data underwent rank-based inverse normal transformation for normalisation. AIRE-DM predictions were adjusted for various covariates using linear regression, including age at the imaging visit, sex, height, BMI, imaging assessment centre, and the first 10 genetic principal components. The UKB participants selected for the genetic analyses were chosen based on European ancestry, SNP missingness rate <10%, absence of sex discrepancies, and exclusion of outliers in terms of heterozygosity or relatedness. ECGs were available for 27,988 individuals. Quality control measures were applied to exclude SNPs with a minor allele frequency <0.1%, genotyping rate <95%, deviation from Hardy–Weinberg equilibrium (p < 1.0 ×10^−8^), or imputation score <0.4 INFO.

The GWAS was performed using the FastGWA MLM implemented by the Genome-wide Complex Trait Analysis (GCTA) software, employing a genetic relationship matrix (GRM) to account for population structure (14). Prior to the analysis, the AIRE-DM score distributions were normalised using rank-based inverse normal transformation. Covariates included in the analysis were age, sex, height, BMI, the UKB assessment centre, and the first 10 genetic principal components. SNPs identified by the conventional genome-wide significance threshold of p-value < 5 ×10^−8^ are reported.

The genomic-relatedness-based restricted maximum likelihood (GREML) analysis, implemented in the GCTA software (15), was used to calculate the genetic variance explained by genome-wide SNPs (SNP-based heritability). Genetic correlation was determined using the bivariate GREML analysis method (16). **Supplementary Figure 9** presents the QQ plots for GWAS summary statistics and gene-based tests.

# Supplementary Tables/Figures

## Supplementary Table 1: Hazard ratios of Cox regression analyses of AIRE-DM score

a) A Cox proportional hazards regression analysis assessing the association between the continuous AIRE-DM score and the risk of developing future T2DM, adjusted for age and sex. The hazard ratio represents the relative change in the risk of future T2DM per one standard deviation (SD) increase in the AIRE-DM score

a)

| **Hazard Ratios from survival analysis of continuous AIRE-DM score, adjusted for age and sex** | | | |
| --- | --- | --- | --- |
| **Cohort** | **HR (AIRE-DM)** | **95% CI** | **P-value** |
| **BIDMC** | 1.74 | 1.68 – 1.79 | < 1e-5 |
| **UKB** | 1.92 | 1.74 - 2.13 | < 1e-5 |
| **ELSA-Brasil** | 1.48 | 1.39 – 1.59 | < 1e-5 |

## Supplementary Figure 1: Calibration of AIRE-DM for the prediction of future incident T2DM in the BIDMC, UKB and ELSA-Brasil cohorts

## Supplementary Figure 2: The VAE convolutional architecture

## Supplementary Figure 3: GWAS QQ plots

# References

1.	Sudlow C, Gallacher J, Allen N, Beral V, Burton P, Danesh J, et al. UK biobank: an open access resource for identifying the causes of a wide range of complex diseases of middle and old age. PLoS Med. 2015;12(3):e1001779.

2.	Fry A, Littlejohns TJ, Sudlow C, Doherty N, Adamska L, Sprosen T, et al. Comparison of Sociodemographic and Health-Related Characteristics of UK Biobank Participants With Those of the General Population. Am J Epidemiol. 2017;186(9):1026-34.

3.	Bai W, Suzuki H, Huang J, Francis C, Wang S, Tarroni G, et al. A population-based phenome-wide association study of cardiac and aortic structure and function. Nature Medicine. 2020;26(10):1654-62.

4.	Ardissino M, McCracken C, Bard A, Antoniades C, Neubauer S, Harvey NC, et al. Pericardial adiposity is independently linked to adverse cardiovascular phenotypes: a CMR study of 42 598 UK Biobank participants. Eur Heart J Cardiovasc Imaging. 2022;23(11):1471-81.

5.	Stabenau HF, Waks JW. BRAVEHEART: Open-source software for automated electrocardiographic and vectorcardiographic analysis. Comput Methods Programs Biomed. 2023;242:107798.

6.	Bang H, Edwards AM, Bomback AS, Ballantyne CM, Brillon D, Callahan MA, et al. Development and Validation of a Patient Self-assessment Score for Diabetes Risk. Annals of Internal Medicine. 2009;151(11):775-83.

7.	Griffin SJ, Little PS, Hales CN, Kinmonth AL, Wareham NJ. Diabetes risk score: towards earlier detection of Type 2 diabetes in general practice. Diabetes/Metabolism Research and Reviews. 2000;16(3):164-71.

8.	Use of Glycated Haemoglobin (HbA1c) in the Diagnosis of Diabetes Mellitus: Abbreviated Report of a WHO Consultation. Geneva: World Health Organization. 2011.

9.	American Diabetes Association. Diagnosis and Classification of Diabetes Mellitus. Diabetes Care. 2010;33(Supplement_1):S62-S9.

10.	Attia ZI, Kapa S, Lopez-Jimenez F, McKie PM, Ladewig DJ, Satam G, et al. Screening for cardiac contractile dysfunction using an artificial intelligence-enabled electrocardiogram. Nat Med. 2019;25(1):70-4.

11.	Sau A, Ibrahim S, Ahmed A, Handa B, Kramer DB, Waks JW, et al. Artificial intelligence-enabled electrocardiogram to distinguish cavotricuspid isthmus dependence from other atrial tachycardia mechanisms(). Eur Heart J Digit Health. 2022;3(3):405-14.

12.	van de Leur RR, Bos MN, Taha K, Sammani A, Yeung MW, van Duijvenboden S, et al. Improving explainability of deep neural network-based electrocardiogram interpretation using variational auto-encoders(). Eur Heart J Digit Health. 2022;3(3):390-404.

13.	Nagueh SF, Smiseth OA, Appleton CP, Byrd BF, Dokainish H, Edvardsen T, et al. Recommendations for the Evaluation of Left Ventricular Diastolic Function by Echocardiography: An Update from the American Society of Echocardiography and the European Association of Cardiovascular Imaging. European Heart Journal – Cardiovascular Imaging. 2016;17(12):1321-60.

14.	Jiang L, Zheng Z, Qi T, Kemper KE, Wray NR, Visscher PM, et al. A resource-efficient tool for mixed model association analysis of large-scale data. Nat Genet. 2019;51(12):1749-55.

15.	Yang J, Benyamin B, McEvoy BP, Gordon S, Henders AK, Nyholt DR, et al. Common SNPs explain a large proportion of the heritability for human height. Nat Genet. 2010;42(7):565-9.

16.	Lee SH, Yang J, Goddard ME, Visscher PM, Wray NR. Estimation of pleiotropy between complex diseases using single-nucleotide polymorphism-derived genomic relationships and restricted maximum likelihood. Bioinformatics. 2012;28(19):2540-2.
